# Identification of early and distinct glioblastoma response patterns treated by boron neutron capture therapy not predicted by standard radiographic assessment using functional diffusion map

**DOI:** 10.1186/1748-717X-8-192

**Published:** 2013-08-01

**Authors:** Ryo Hiramatsu, Shinji Kawabata, Motomasa Furuse, Shin-Ichi Miyatake, Toshihiko Kuroiwa

**Affiliations:** 1 Department of Neurosurgery, Osaka Medical College, 2-7 Daigaku-machi, Takatsuki City, Osaka 569-8686, Japan

**Keywords:** ADC, BNCT, Diffusion MRI, fDM, GB

## Abstract

**Background:**

Radiologic response of brain tumors is traditionally assessed according to the Macdonald criteria 10 weeks from the start of therapy. Because glioblastoma (GB) responds in days rather than weeks after boron neutron capture therapy (BNCT) that is a form of tumor-selective particle radiation, it is inconvenient to use the Macdonald criteria to assess the therapeutic efficacy of BNCT by gadolinium-magnetic resonance imaging (Gd-MRI). Our study assessed the utility of functional diffusion map (fDM) for evaluating response patterns in GB treated by BNCT.

**Methods:**

The fDM is an image assessment using time-dependent changes of apparent diffusion coefficient (ADC) in tumors on a voxel-by-voxel approach. Other than time-dependent changes of ADC, fDM can automatically assess minimum/maximum ADC, Response Evaluation Criteria In Solid Tumors (RECIST), and the volume of enhanced lesions on Gd-MRI over time. We assessed 17 GB patients treated by BNCT using fDM. Additionally, in order to verify our results, we performed a histopathological examination using F98 rat glioma models.

**Results:**

Only the volume of tumor with decreased ADC by fDM at 2 days after BNCT was a good predictor for GB patients treated by BNCT (P value = 0.022 by log-rank test and 0.033 by wilcoxon test). In a histopathological examination, brain sections of F98 rat glioma models treated by BNCT showed cell swelling of both the nuclei and the cytoplasm compared with untreated rat glioma models.

**Conclusions:**

The fDM could identify response patterns in BNCT-treated GB earlier than a standard radiographic assessment. Early detection of treatment failure can allow a change or supplementation before tumor progression and might lead to an improvement of GB patients’ prognosis.

## Background

Surgery followed by radiation therapy is still the standard treatment for glioblastoma (GB). The addition of temozolomide (TMZ) chemotherapy to the standard treatment has significantly increased the proportion of patients who survive longer than 2 years [1]. However, additional progress is needed, as almost half of GB patients do not survive the first year after diagnosis.

Boron neutron capture therapy (BNCT) has been developed in the hope of achieving a breakthrough in GB treatment [2,3]. BNCT is a form of tumor-selective particle radiation therapy. We have applied BNCT to over 80 GB patients and have reported its survival benefit [4]. Additionally, a phase II multicenter clinical trial of BNCT is currently underway in Japan. In our substantial experience of clinical BNCT, we have frequently experienced dramatic reductions in enhanced lesion size on gadolinium-magnetic resonance imaging (Gd-MRI) obtained 2 to 7 days after BNCT [2,3]. Assessment of radiation and chemotherapy efficacy for GB patients is traditionally accomplished by measuring changes in contrast enhancement of tumors at 10 weeks from the start of therapy using Gd-MRI, using the so-called Macdonald criteria [5]. The Macdonald criteria guide standard radiographic assessments, and have been correlated with survival [5-7]. However, because GB responds in days rather than weeks after BNCT, it is inconvenient to use the Macdonald criteria (including the conventional timing) to assess the therapeutic efficacy of BNCT by Gd-MRI.

On the other hand, the current standard treatment for GB patients, combined chemo-irradiation with TMZ, may induce pseudoprogression in 20–30% of cases [8], defined as an increase of contrast enhancement and/or edema on MRI without true tumor progression [9]. Also, full-blown radiation necrosis may be more frequent after combined chemo-irradiation. Pseudoresponse - namely, a decrease in contrast enhancement of brain tumors on MRI without a decrease of tumor activity - is frequent after treatment with vascular endothelial growth factor receptor signalling pathway inhibitors. Just as it is difficult to evaluate response patterns of GB treated by BNCT, so also cases with pseudoprogression, radiation necrosis, or pseudoresponse are difficult to assess using standard radiography because of changes in contrast enhancement that do not reflect tumor activity.

Diffusion MRI, which measures the random (Brownian) motion of water, has been proposed as an early biomarker for tumor response that does not rely on the measurement of contrast enhancement [10], and has been evaluated in preclinical [11,12] and clinical studies [13-15]. Diffusion MRI measurements are sensitive and can be used to detect and quantify tissue water diffusion values, which have been proposed to be related to the ratio of intracellular water to extracellular water; thus, changes in apparent diffusion coefficient (ADC) are inversely correlated with changes in cellularity. In this scenario, increases in ADC would reflect an increase in the mobility of water, either through the loss of membrane integrity or an increase in the proportion of total extracellular fluid with a corresponding decrease in cellular size or number, as seen with necrosis or apoptosis. In contrast, decreases in ADC reflect a decrease in free extracellular water, either through an increase in total cellular size or number, as can be seen with tumor progression or tumor cell swelling [16].

Functional diffusion map (fDM) was developed to take advantage of these principles on a voxel-by-voxel approach, and have proven to be a powerful tool for predicting the effect of chemotherapy and radiotherapy [10,15,17]. An increased ADC has been shown to correlate with a decrease in cellularity as a result of successful treatment [11,18] and/or radiation necrosis [18]. Other than time-dependent changes of ADC, fDM could automatically assess minimum (Min)/maximum (Max) ADC, Response Evaluation Criteria In Solid Tumors (RECIST), and the volume of enhanced lesions in response to BNCT over time.

In the current study, the usefulness of fDM as a predictive biomarker for GB patients treated with radiochemotherapy was reported [14,15]. There are no reports about the usefulness of fDM for GB treated by BNCT. In order to verify the usefulness of fDM for GB patients treated by BNCT, we assessed 17 GB patients treated by BNCT with fDM at 2 days after BNCT and examined a relationship between all the above factors analyzed by fDM (time-dependent changes of ADC, Min/Max ADC, RECIST, and the volume of enhanced lesions) and prognosis of GB patients treated by BNCT. Additionally, we treated F98 rat glioma models with BNCT and compared brain sections of the BNCT group with the untreated group using hematoxylin-eosin (H & E) staining.

## Methods

### Patient population

We performed a retrospective investigation of clinical BNCT to evaluate the effects of therapy and adverse events. From June 2003 to December 2007, we treated a total of 61 GB patients using BNCT. Because 17 of these 61 GB patients (8 females; 9 males) had diffusion MRI at pre- and post-BNCT and had contrast enhancement volumes over 0.7 cm^3^ on Gd-MRI, we were able to assess them using fDM. Ten patients were newly diagnosed with GB and 7 patients were recurrent GB cases. The average age was 56.7 years (36–74 years). The average survival time from BNCT was 14.5 months (7.2 - 45.9 months). The average volume of contrast enhancement on Gd-MRIs was 18.8 cm^3^ (0.7 - 51.4 cm^3^).

### Our treatment for GB patients and boron neutron capture therapy protocol

Our treatment for GB patients was surgical resection as much of the tumor as possible, followed by BNCT. Our BNCT protocol was as follows:

Twelve hours before the neutron irradiation, the patients were administered 100 mg/kg or none of sodium borocaptate intravenously for 1 hour. Boronophenylalanine (BPA) of 250 mg/kg was infused continuously to the patients for 1 hours or 700 mg/kg was infused continuously to the patients for 6 hours before the irradiation, and they were positioned for neutron irradiation in the atomic reactor (Kyoto University Research Reactor [KUR] or Japan Atomic Energy Agency Research Reactor 4). Just after termination of continuous BPA infusion for 6 hours, neutrons were irradiated. Between June 2003 and December 2006, no chemotherapy was applied for any of the patients until the tumor progression was confirmed histologically or by ^18^F-BPA-positron emission tomography [19]. This protocol was approved by the Ethical Committee of Osaka Medical College and also by the Committee for Reactor Medicine in KUR. The indication of BNCT for each candidate was discussed by the latter committee.

### MRI examinations

All patients underwent pre-BNCT MRI within 20 days before BNCT and underwent post-BNCT MRI at 2 days after BNCT. MRI examinations were composed of T1-weighted images MRI, T2-weighted images, fluid attenuation inversion recovery (FLAIR) images, Gd-T1-weighted images and diffusion images. MRI was performed on a 1.5-T MRI system (GE; Wisconsin, Milwaukee, USA). MRI sequences included T1-weighted images (TE/TR = 9 ms/2500 ms, slice thickness = 5 mm with 2.5 mm interslice distance, number of excitations [NEX] = 1, matrix size = 256 × 224, and field of view [FOV] = 24 cm), T2-weighted images (TE/TR = 103 ms/2500 ms, slice thickness = 5 mm with 2.5 mm interslice distance, NEX = 1, matrix size = 320 × 192, and FOV = 24 cm), and FLAIR images (inversion time = 2200 ms, TE/TR = 116.7 ms/8800 ms, slice thickness = 5 mm with 2.5 mm interslice distance, NEX = 1, matrix size = 256 × 192, and FOV = 24 cm). In addition, Gd-T1-weighted images (axial: TE/TR = 9 ms/400 ms, slice thickness 5 mm with 2.5 mm interslice distance, NEX = 1, a matrix size of 256 × 224, and FOV = 24 cm) were acquired after contrast injection (Magnevist; Berlex; 0.1 mmol/kg) (Table 1).

**Table 1:** Summarizing the details of MRI sequences

**MRI Sequences**	**T1**	**T2**	**FLAIR**	**DWI**	**Gd-T1**
TR (ms)	2500	4000	8800	4500	400
TE (ms)	9	103	116.7	67.7	9
inversion time (ms)			2200		
FOV* (cm)	24	24	24	24	24
slice thickness (mm)	5	5	5	5	5
interslice distance (mm)	2.5	2.5	2.5	0	2.5
frequency matrix	256	320	256	128	256
phase matrix	224	192	192	192	224
NEX**	1	1	1	2	1
scan time (s)	107	144	121	64	94

*FOV = field of view **NEX = number of excitations.

### Diffusion MRI

Diffusion MRI was collected with TE/TR = 79.3 ms/6400 ms, NEX = 1, slice thickness = 5 mm with 0 mm interslice distance, matrix size = 128 × 192 and a FOV = 24 cm. ADC images were calculated from acquired DWIs with b = 1000 s/mm^2^ and b = 0 s/mm^2^ images (Table 1). Diffusion images for the three orthogonal directions were combined to calculate an ADC map [20].

### fDM analysis

**Figure 1:**
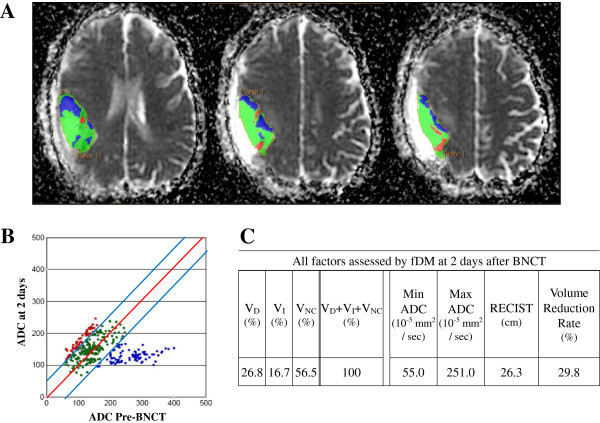
**Representative case: Regions of interests were drawn for tumor image by using anatomical images (A).** Red voxels represent areas within the tumor where ADC increased (> 55 × 10^-5^ mm^2^/sec); blue voxels represent decreased ADC (< 55 × 10^-5^ mm^2^/sec), and green voxels represent no change. These thresholds represent the 95% confidence intervals for change in ADC for the uninvolved cerebral hemisphere **(B)**. V_D_, V_I_, V_NC_, Min/Max ADC, RECIST, and the volume of enhanced lesions on Gd-MRI over time showed 26.8%, 16.7%, 56.5%, 55.0 10^-5^ mm^2^/sec, 251.0 10^-5^ mm^2^/sec, 26.3 cm, and 29.8% at 2 days after BNCT, respectively **(C)**.

All MRIs were spatially co-registered using the pre-BNCT Gd-MRI as the reference dataset. This step allowed all images of a given patient to be viewed and analyzed from a fixed frame of reference. The co-registration was performed using a “mutual information for automatic multimodality image fusion” (MIAMI FUSE) algorithm [21]. After this co-registration, brain tumors were manually segmented on the Gd-MRIs by a neurosurgeon (R. H.). These segmentations were copied into the contemporary diffusion MRIs and were analyzed using a voxel-by-voxel approach [17,22]. A minimum of 0.7 cm^3^ of tumor on Gd-MRI was necessary for eligibility. If a resection cavity was present, it wasn’t included within the regions of interest if circumscribed by contrast enhancement. Only voxels present in both the pre-BNCT and post-BNCT tumor volumes were included for fDM analysis. Individual voxels were stratified into three categories based on the change in ADC from the pre-BNCT scan to each time point. Red voxels represent areas within the tumor where ADC increased (> 55 × 10^-5^ mm^2^/sec); blue voxels represent decreased ADC (< 55 × 10^-5^ mm^2^/sec), and green voxels represent no change (Figure 1). These thresholds represent the 95% confidence intervals for change in ADC for the uninvolved cerebral hemisphere [17]. The percentages of the tumor within these three categories were calculated as V_I_, V_D_, and V_NC_, respectively. Other than time-dependent changes of ADC, fDM could automatically assess Min/Max ADC, RECIST, and the volume of enhanced lesions in response to BNCT over time. These analyses were performed using fDM analysis software (*I*-*Response*™-*1*.*0*, Cedara software; Ontario Canada).

### Representative case

This patient was newly diagnosed GB with 23.2 months of patients’ survival time after BNCT. Depicted images are single slices of Gd-MRI scans at 2 days after BNCT with a pseudocolor overlay of the fDM. Red voxels indicate regions with a significant rise in ADC at 2 days after BNCT compared with pre-BNCT, green regions had no changed ADC, and blue voxels indicate areas of significant decline in ADC (Figure 1A). The scatter plots display data for the entire tumor volume and not just for the depicted slice at 2 days after BNCT, with ADC of the pre-BNCT on the *x*-axis and ADC at 2 days after BNCT on the *y*-axis. The central red line represents unity, and the flanking blue lines represent the 95% confidence interval (CI) (Figure 1B). Other than time-dependent changes of ADC, fDM can automatically assess maximum/minimum ADC, RECIST, and the volume of enhanced lesions on Gd-MRI over time (Figure 1C).

### Correlation with all factors assessed by fDM and survival time after BNCT

All factors assessed by fDM are composed of V_I_, V_D_, V_NC_, Min/Max ADC, RECIST, and the volume reduction rate of enhanced lesions. The end point in this study was a survival time after BNCT. Survival analysis utilized log-rank and wilcoxon test. Statistical analysis utilized JMP® Pro 10 (SAS Institute Inc., Cary, NC, USA).

### Tumor models

F98 rat glioma cells produce infiltrating tumors in the brains of Fischer rats [23]. The tumors have been shown to be refractory to a number of treatment modalities, including radiation therapy [24]. Based on their *in vivo* histology, the F98 rat glioma cells have been characterized as anaplastic or undifferentiated glioma [25]. In the present study, F98 rat glioma cells were kindly obtained from Prof. Barth (Department of Pathology, the Ohio State University, Columbus, OH, USA). They were routinely cultivated in our laboratory in Dulbecco’s Modified Eagle Medium supplemented with 10% fetal bovine serum and penicillin at 37°C in an atmosphere of 5% CO_2_. All the materials for the culture medium were purchased from Gibco Invitrogen (Grand Island, NY, USA). Male Fischer rats weighing 200–250 g were anesthetized with an intraperitoneal injection of Nembutal (50 mg/kg) and placed in a stereotactic frame (Model 900, David Kopf Instruments, Tujunga, CA, USA). A midline scalp incision was made and the bregma was identified. A 1mm burr hole was made in the right frontal region of the skull and a 22-gauge needle attached to a 25-μl syringe was inserted into the caudate nucleus using the same stereotactic coordinates, with the needle tip inserted 5 mm into the dura. An injection of 10^3^ F98 rat glioma cells in 10 μl of serum free medium was administered at a rate of 1 μl/min. After the infusion, the needle was left in place for 3 min and the burr hole was then covered with bone wax.

### Histopathological examination

At 2 weeks after implantation, the BNCT group was administered 250 mg/kg body weight of BPA intravenously. An hour and a half after BPA injection, only the BNCT group was irradiated with neutrons at KUR during 1 hour. All rats of both the BNCT group and the untreated group were euthanized by isoflurane 16 days after implantation (i.e., 2 days after BNCT for the BNCT group). The rats were perfused and fixed by 10% formalin; then the brains were dehydrated and embedded in paraffin. The 4-μm sections were stained with hematoxylin and eosin (H & E) for histopathological investigation. We compared sections of the BNCT group with the untreated group using a light microscope (ECLIPSE80i, Nikon, Japan).

## Results

### MRI examination

In our study, pre-BNCT MRI was performed at 7.9 ± 5.0 (1–20) days before BNCT, and post-BNCT MRI was performed at 2.5 ± 1.6 (1–8) days after BNCT.

### Overall survival and survival time after BNCT of all patients

**Figure 2:**
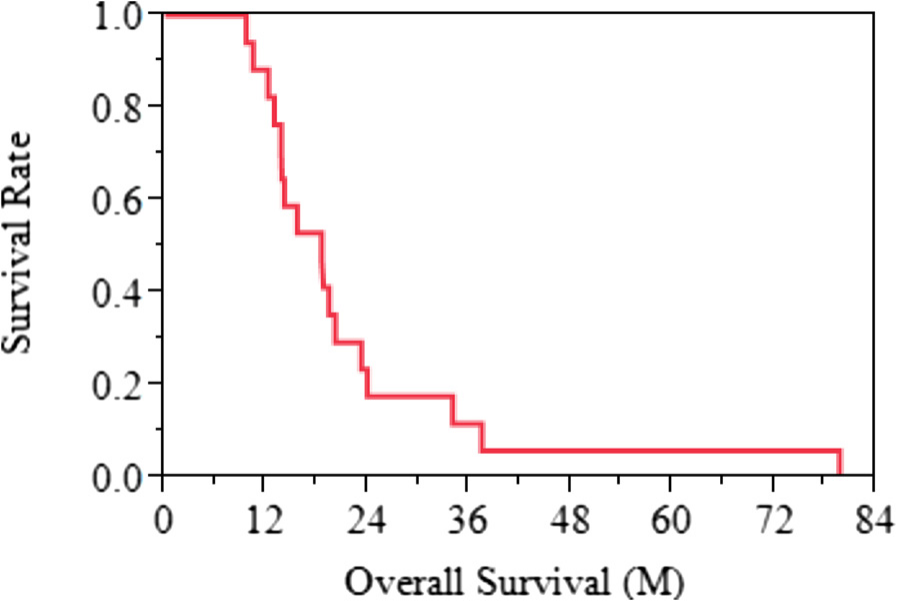
Overall survival of all patients: Median overall survival was 18.5 months (95% CI; 12.9 - 23.2 months).

**Figure 3:**
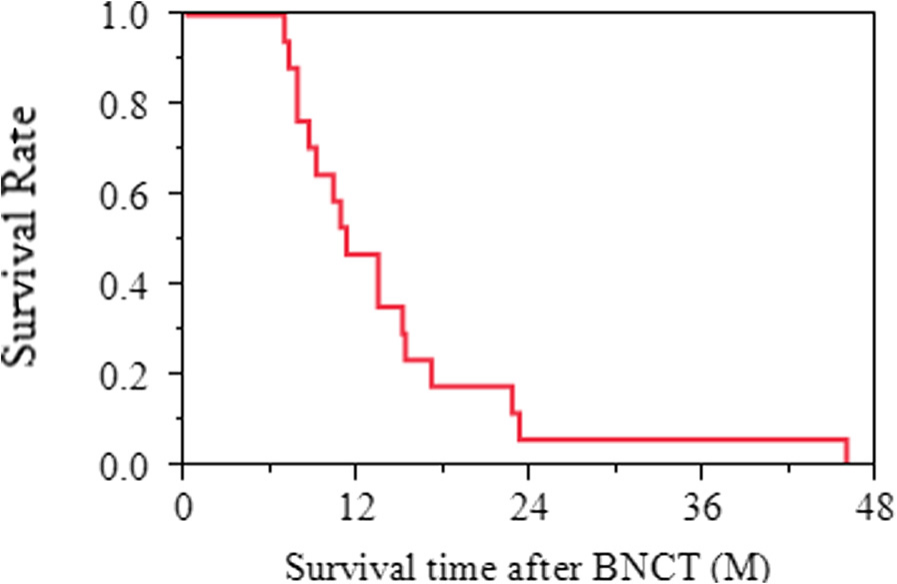
Survival time after BNCT of all patients: Median survival time after BNCT was 11.2 months (95% CI; 7.8 - 15.3 months).

Median overall survival was 18.5 months (95% CI; 12.9 - 23.2 months) (Figure 2) and median survival time after BNCT was 11.2 months (95% CI; 7.8 - 15.3 months) (Figure 3).

### Correlation with all factors assessed by fDM and survival time after BNCT

**Figure 4:**
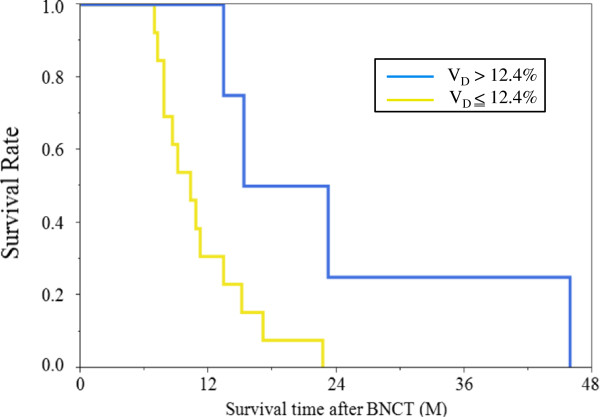
Survival analysis of V_D_ at 2 days after BNCT: V_D_ greater than 12.4% at 2 days after BNCT was good response for BNCT (median survival = 19.3 months; 95% CI = 13.4 - 45.9 months) and V_D_ 12.4% or less at 2 days after BNCT was nonresponse for BNCT (median survival = 10.3 months; 95% CI = 7.8 - 13.4 months).

**Figure 5:**
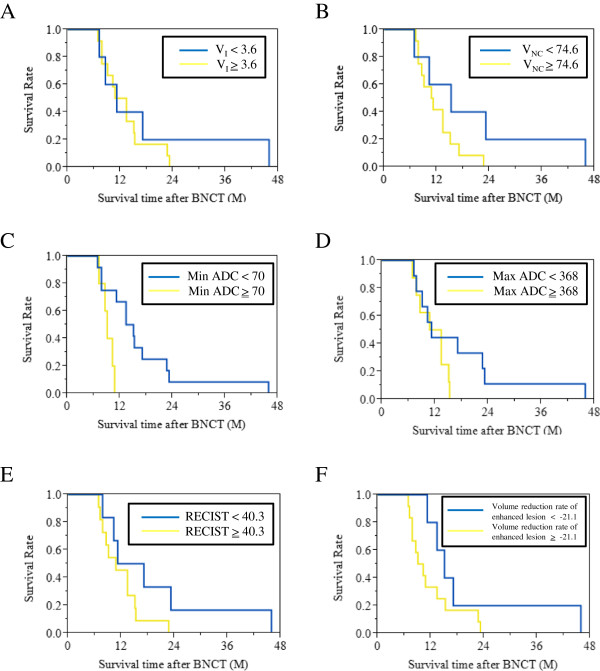
**Survival analysis except for V_D_ at 2 days after BNCT: V_I_, V_NC_, Max ADC, RECIST, and the volume reduction rate of enhanced lesions at 2 days after BNCT (A, B, D, E, and F, respectively) had no correlation with patients’ survival time after BNCT using log-rank test and wilcoxon test.** Min ADC at 2 days after BNCT **(C)** showed a significant difference using log-rank test and wilcoxon test. However, Min ADC showed over-lap in 95% CI (Table 2).

V_D_ and Min ADC at 2 days after BNCT showed a significant difference using log-rank test and wilcoxon test. However, Min ADC showed over-lap in 95% CI. On the other hand, V_D_ showed no over-lap in 95% CI (Table 2). V_D_ greater than 12.4% at 2 days after BNCT was good response for BNCT (median survival = 23.2 months; 95% CI = 13.4 - 45.9 months) and V_D_ 12.4% or less at 2 days after BNCT was nonresponse for BNCT (median survival = 10.3 months; 95% CI = 7.8 - 13.4 months) (Figure 4). Survival analysis of V_D_ showed a significant difference (P value = 0.022 by log-rank test and 0.033 by wilcoxon test). However, V_I_, V_NC_, Max ADC, RECIST, and the volume reduction rate of enhanced lesions at 2 days after BNCT had no correlation with patients’ survival time after BNCT (Figure 5, Table 2).

**Table 2:** Survival analysis of all factors assessed by fDM

		**All factors assessed by fDM**													
		**V**_**D **_**(%)**		**V**_**I **_**(%)**		**V**_**NC **_**(%)**		**Min ADC (10**^**-5 **^**mm2/sec)**		**Max ADC (10**^**-5 **^**mm2/sec)**		**RECIST (cm)**		**Volume reduction rate of enhanced lesion (%)**	
		> 12.4	≤ 12.4	< 3.6	≥ 3.6	< 74.6	≥ 74.6	< 70	≥ 70	< 368	≥ 368	< 40.3	≥ 40.3	< −21.1	≥ −21.1
**Median time** (M)		19.3	10.3	11.2	12.1	15.3	11	14.3	9.1	11.2	12.1	14.2	10.8	15.1	9.7
**95% CI**		13.4-	7.8-	7.2-	7.8-	6.9-	7.8-	7.8-	7.2-	7.2-	6.9-	7.8-	7.2-	11.2-	7.2-
(M)		45.9**	13.4**	45.9	15.3	45.9	15.1	22.7**	10.8**	23.2	15.1	45.9	15.1	45.9	15.3
**p value**	**log-rank**	0.022*		0.521		0.128		0.011*		0.176		0.118		0.143	
	**wilcoxon**	0.033*		0.834		0.35		0.045*		0.413		0.223		0.083	

*V_D_ and Min ADC at 2 days after BNCT showed a significant difference using log-rank test and wilcoxon test. **However, Min ADC showed over-lap in 95% CI. On the other hand, V_D_ showed no over-lap in 95% CI.

### Histopathological examination

**Figure 6:**
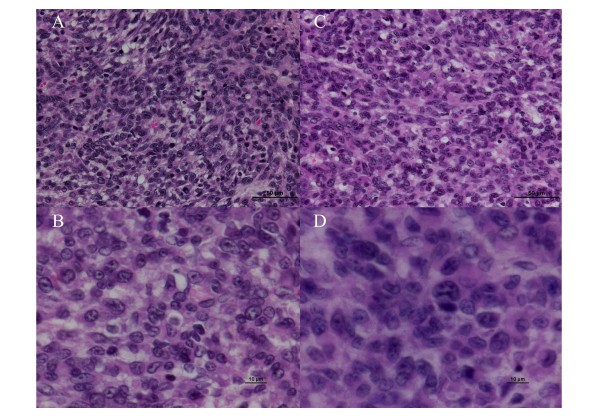
**Histopathological examination: Tumor cells in the BNCT group showed the cell swelling of both the nuclei and the cytoplasm (C and D) compared with the untreated group (A and B) at 16 days after the implantation (i.e., at 2 days after BNCT for the BNCT group).** (**A** and **C** were at 400-fold. **B** and **D** were 1000-fold magnification).

Tumor cells in the BNCT group showed swelling of both the nuclei and the cytoplasm compared with the untreated group at 16 days after implantation (i.e., 2 days after BNCT for the BNCT group) (Figure 6).

## Discussion

In 1990, Macdonald *et al*. reported criteria for response assessment in glioma [5]. Although these criteria have limitations, they have become widely accepted. However, recent observations have revealed the fundamental limitations of the Macdonald criteria [26,27]. One limitation of the Macdonald criteria is the extended time required to detect change [5,28,29], about 8 to 10 weeks. Another is the discrepancy between contrast enhancement and tumor activity. At the core of Macdonald criteria are changes in contrast enhancement, and all too often, the contrast enhancement of high-grade tumors is perceived as a measure of tumor activity. However, contrast enhancement is nonspecific and primarily reflects a disrupted blood–brain barrier. Contrast enhancement can be influenced by changes in corticosteroid dose and radiologic technique [9,30]. Contrast enhancement can also be induced by a variety of nontumoral processes: inflammation, seizure activity, postsurgical changes, pseudoprogression, radiation necrosis, and pseudoresponse [9,31]. As a result, changes in contrast enhancement cannot be equated with changes in tumor size or tumor growth/activity.

Recently, several novel imaging methods—positron-emission tomography, single-photon emission computerized tomography, MR spectroscopy, and diffusion MRI—have been evaluated for their ability to assess early therapeutic responses independently of late changes in enhanced tumor volume [32,33]. Diffusion MRI detection of cancer treatment response was first successfully reported in a rodent brain tumor model treated with chemotherapy. Additionally, Diffusion MRI has been evaluated in preclinical [11,12,34] and clinical studies [13,34,35]. In 2008, Hamstra *et al*. assessed high-grade glioma with functional diffusion map. They reported that the volume of tumor with increased diffusion by fDM at 3 weeks after the start of radiation therapy was the strongest predictor of patient survival at 1 year [10].

In our study, V_D_ at 2 days after BNCT was the strongest predictor of GB patients’ survival time after BNCT. V_D_ (= the volume of the voxels with decreased ADC compared with pre-BNCT by fDM) indicates that extracellular free water is relatively decreased for the highest volume of tumor cells. So, this appearance is attributed to tumor progression or tumor cell swelling as previously mentioned in the ***Background***. In our study, day 2 V_D_ was a good predictor for GB patients treated by BNCT. We attributed this higher V_D_ to tumor cell swelling rather than tumor progression. In fact, our histopathological study detected tumor cell swelling in the BNCT group compared with the untreated group at 16 days after the implantation (i.e., at 2 days after BNCT for the BNCT group) (Figures 5 and 6). Others have reported tumor cell swelling in the acute stage after BNCT. Kato *et al*. reported the pathological changes of oral squamous cell carcinoma at an early stage after BNCT using nude mouse subcutaneous models. They compared a BNCT group with an untreated group using pathological analysis at 1, 2, and 7 days after BNCT. Compared to the untreated group, oral squamous cell carcinoma in the BNCT group at all early stages showed tumor cell swelling on the H & E stained nude mouse brain sections [36]. Nakagawa *et al*. reported early effects of BNCT on C6 rat glioma models. They compared a BNCT group with an untreated group using pathological analysis at 4days after BNCT. Compared to the untreated group, C6 rat glioma cell in the BNCT group showed cell swelling on the H & E stained rat brain sections [37].

## Conclusions

Our study proved that fDM was useful for evaluating the therapeutic efficacy of BNCT in GB patients treated by BNCT. Additionally, fDM could identify response patterns in BNCT-treated GB earlier than a standard radiographic assessment. Early detection of treatment failure can allow a change or supplementation before tumor progression and might lead to an improvement of GB patients’ prognosis.

## Abbreviations

ADC: Apparent diffusion coefficient; BNCT: Boron neutron capture therapy; BPA: Boronophenylalanine; CI: Confidence interval; fDM: functional diffusion map; FLAIR: Fluid attenuation inversion recovery; FOV: Field of view; GB: Glioblastoma; Gd: Gadolinium; H & E: Hematoxylin and Eosin; KUR: Kyoto university research reactor; Max: Maximum; Min: Minimum; MRI: Magnetic resonance imaging; NEX: Number of excitations; RECIST: Response evaluation criteria in solid tumors; TMZ: Temozolomide.

## Competing interests

The authors declare that they have no competing interests.

## Authors' contributions

RH carried out all the animal study and the statical analysis, and drafted the manuscript. SK conceived of the study, and participated in its design and coordination and helped to draft the manuscript. MF, S-IM, and TK participated in its design and coordination and helped to draft the manuscript. All authors read and approved the final manuscript.
